# LEAVES (optimizing the mentaL health and resiliencE of older Adults that haVe lost thEir spouSe via blended, online therapy): Proposal for an Online Service Development and Evaluation

**DOI:** 10.2196/19344

**Published:** 2020-09-08

**Authors:** Lex van Velsen, Miriam Cabrita, Harm op den Akker, Lena Brandl, Joana Isaac, María Suárez, Afonso Gouveia, Rute Dinis de Sousa, Ana Maria Rodrigues, Helena Canhão, Nathaniel Evans, DELA Natura- En Levensverzekeringen NV, Marije Blok, Carlos Alcobia, Jeannette Brodbeck

**Affiliations:** 1 eHealth group Roessingh Research and Development Enschede Netherlands; 2 Biomedical Signals and Systems Group University of Twente Enschede Netherlands; 3 Unidade Local de Saúde do Baixo Alentejo Beja Portugal; 4 Comprehensive Health Research Center NOVA Medical School NOVA University of Lisbon Lisbon Portugal; 5 Nothing AG Bern Switzerland; 6 See Acknowledgments; 7 National Foundation for the Elderly Amersfoort Netherlands; 8 Department of Sociology Vrije Universiteit Amsterdam Netherlands; 9 Sensing Future Technologies Coimbra Portugal; 10 School of Social Work FHNW University of Applied Sciences and Arts Northwestern Switzerland Olten Switzerland; 11 Department for Clinical Psychology and Psychotherapy University of Bern Bern Switzerland

**Keywords:** eHealth, grief, bereavement, widowhood, aged, resilience, telemedicine, mental health

## Abstract

**Background:**

Loss of a spouse is a frequent occurrence in later life. While most older adults successfully process this loss and will return to a normal life, about 10% of the individuals are unable to cope, and progress to prolonged grief (PG). PG, in turn, can result in mental and physical problems including poor sleep, cardiovascular problems, depression, and suicidal tendencies.

**Objective:**

LEAVES (optimizing the mentaL health and resiliencE of older Adults that haVe lost thEir spouSe via blended, online therapy) is an online bereavement program that will support the prevention and treatment of PG, so that elderly mourners can continue to lead an active, meaningful, and dignified life. LEAVES will cater to secondary end users (eg, family, informal caregivers) by reducing stress.

**Methods:**

LEAVES will help older adults to process the loss of a spouse in an online environment, which consists of (1) an existing online grief self-help program LIVIA, (2) the Before You Leave program that allows for storing personal memories, (3) a virtual agent platform, and (4) an accessible front-end design. LEAVES can detect persons at risk for complications, reveal negative trends in their emotional life, and act to counter such trends. The service relies on online support whenever possible but is blended with telephone or face-to-face counseling when necessary.

**Results:**

The project will take place between February 2020 and January 2023 and includes a real-life evaluation in which 315 end users will use the service across 3 countries (the Netherlands, Portugal, and Switzerland). The evaluation of LEAVES will focus on clinical effect, its business case, and technology acceptance. The results will pave the way for smooth integration into existing care paths and reimbursement schemes.

**Conclusions:**

The LEAVES service aims to soften the mourning process, prevents depression or social isolation, strengthens widow(er)s resilience and well-being, and quickens one’s return to societal participation.

**International Registered Report Identifier (IRRID):**

DERR1-10.2196/19344

## Introduction

### Background

Mary has been married to Frank for 30 years. Frank is now facing the end of his life, due to progressing cancer. Frank passes away and Mary is left to herself. Mary was always focused on her close family, but now that Frank is gone and her only daughter Lisa has returned to London, she will have to face her mourning process and adaptation to a new life by herself.

When older adults lose their partner, they often lose the most important person in their life. Most mourners grieve for a period and then find a way to cope with their loss. Some mourners, however, develop severe or persistent grief symptoms, with a clinical diagnosis of a prolonged grief disorder (as defined in International Classification of Diseases [ICD]-11) or a persistent complex bereavement disorder (as defined in Diagnostic and Statistical Manual of Mental Disorders [DSM]-5). Within the context of LEAVES (optimizing the mentaL health and resiliencE of older Adults that haVe lost thEir spouSe via blended, online therapy), we will use the term prolonged grief (PG). PG is the “failure to return to pre-loss levels of performance or states of emotional wellbeing” [1] and commences when grief-related symptoms are still present after 6 months. These symptoms consist of separation distress (eg, yearning, intensive sorrow, and emotional pain), reactive distress (eg, difficulties accepting the loss, self-blame, and avoidance of reminders of the loss), and social/identity disruption (eg, loneliness, meaninglessness, and role confusion), which can lead to impairments in important life domains. Comorbidity of PG with depression, post-traumatic stress disorder, and suicidal ideation is high [2]. Bereavement in later life has also been associated with physical problems and risk behaviors including involuntary weight loss, poor sleep, change in smoking and alcohol habits, chronic pain, inflammation and cardiovascular risks, as well as increased mortality [3-6]. PG has an estimated prevalence of 10% among the general elderly population [7], with the estimated rates differing per gender. For example, a German population-based study found prevalence rates of complicated grief of 9.6% for female and 2.7% for male older adults [8]. A study conducted on a large sample of Dutch older adults found that of those experiencing grief, 25.4% develop PG [9].

The course of the grieving period and whether or not a person develops PG depend on several factors, such as the mourner’s resilience and their social network [10]. Although it is possible to predict the risk for one to develop PG [11], screening is not a common practice. Furthermore, the development of PG often goes unnoticed by the widow/widower, who then also does not realize that she or he can benefit from help [12]. This has led to a situation in which older adults are unaware of their own needs, do not seek help, and do not receive the care they need.

A wide range of interventions are available for either preventing or treating PG including support groups, writing exercises, and individual psychological counseling. Internet-based options have also started to emerge (eg, [13-16]). Such interventions mostly consist of writing exercises with minimal therapist involvement. A recent evaluation of an internet-based, preventive intervention shows a moderate to large reduction in the duration of grief, depression, anxiety, and psychopathological distress [17]. In general, however, the evidence on the effect of online preventive interventions remains inconclusive.

In this proposal and the upcoming project LEAVES, we will develop, implement, and evaluate an online bereavement support program that will support the prevention and treatment of PG. In parallel, we will develop an exploitation strategy for the service, so that it can persist on a sustainable model following project completion.

### LEAVES Online Bereavement Support Service

Mary receives an email from LEAVES. LEAVES offers her the option to be guided throughout her mourning process.

The LEAVES service will provide support to older adults who have lost their spouse, helping them cope with their grief, by preventing PG or treating PG once detected. Ultimately, the aim is to prevent depression or social isolation, strengthen resilience and well-being, and accelerate one’s return to society. LEAVES helps older adults to process the loss of a spouse in an empathic and caring online environment. It can detect persons at risk for complications following loss of a spouse, reveal negative trends in the emotional life of the widow or widower, and intervene to counter such trends. The service will rely on online treatment whenever possible but will be blended with telephone or face-to-face counseling when necessary.

**Figure 1:**
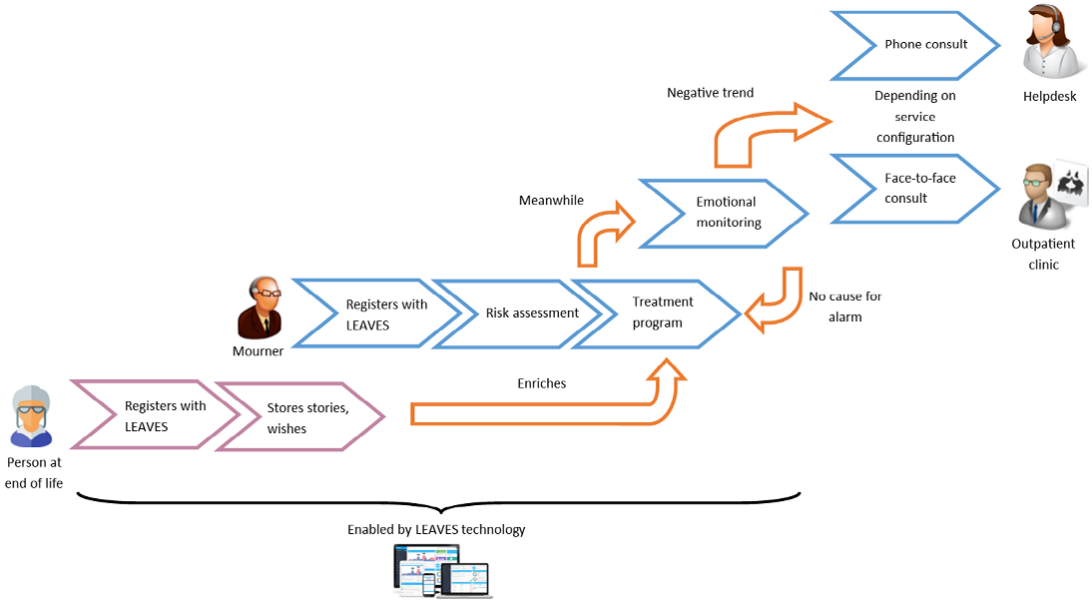
LEAVES (optimizing the mentaL health and resiliencE of older Adults that haVe lost thEir spouSe via blended, online therapy) value chain.

The LEAVES value chain (Figure 1) begins when a person at the end of their life is presented with the LEAVES service, for example, by an elderly association (as a service to their members) or a funeral home (as part of their service package). Once the person using the service passes away, the mourning spouse is automatically invited to the service. Alternatively, those in mourning can register for the service without prior participation of the deceased spouse. After registering with the service via the LEAVES website, a user profile is created for each individual. This profile includes demographics (for personalizing the service) and a risk assessment for developing PG based on an online survey founded upon scientific risk prediction models. The mourner then enters the treatment pipeline, which is based on the LIVIA program [17,18]. LIVIA focuses on guiding mourners during marital bereavement via a self-service, online program. Online treatment is supported by stories and memories, provided by the person who passed away via the Before You Leave service [19]. To facilitate the interaction between technology and mourner, the LEAVES service will make use of a conversational agent-based interaction paradigm, in which the user interacts through natural language with a virtual on-screen character, an approach that is gaining popularity in health-related applications (eg, [20,21]). Using this technology, a caring, empathic, and individually tailored online environment will be developed to foster trust and compliance. The mourner’s emotional state will be constantly monitored both by assessing the user’s responses during dialogue interactions with the virtual agent and by experience sampling methods (ie, short, periodic Q&A modules), offered via tablet or mobile platforms. If any negative trend is observed indicating potential risk for PG, the mourner will be directed toward LEAVES’ offline services (eg, phone helpdesk, face-to-face meetings). The service will be developed around the notion that *the user will be helped online when possible, but offline when necessary*.

The LEAVES service focusses on the following target groups:

- Primary end users: Older adults (65+) who have recently lost their spouse. The service will enable them to properly cope with the associated life changes.
- Secondary end users: Those close to the mourning spouse (eg, children, neighbors, and friends). They are concerned about the mourner’s situation but may find it hard to estimate his or her emotional state if the mourner remains emotionally closed about the subject. LEAVES can assist by encouraging transparency in the situation.
- Secondary end users: Deceased spouse. Before passing away, one may be concerned about the future of their spouse and his/her ability to cope with the new situation. LEAVES allows them to store their stories and wishes, thereby creating a lasting memory that will ease the mourning process of those left behind.
- Secondary end users: Clinical professionals. They will receive fewer patients suffering from PG. Those who do suffer from PG will seek assistance at an earlier stage.
- Lead users: Undertakers. They offer the service as part of their service package, thereby gaining a competitive advantage.
- Lead users: Elderly associations. They can offer the service as part of their services for their members.

### Consortium

The project consortium consists of a collection of organizations including research, end user, and clinical organizations; small- and mid-sized enterprises; and a large enterprise. The project is led by Roessingh Research and Development (RRD). Service model design and visual design are mainly being taken care of by The Dutch National Foundation for the Elderly (NFE) and the Swiss SME Nothing AG (NTH). Technology development is being led by RRD. RRD has created a solid foundation for an electronic health (eHealth) platform and AI dialogue systems in numerous European and commercial projects. NTH will complement these skills with their functional front-end and graphical user interface design skills. The University of Bern, Switzerland (UoB), the School of Social Work of the University of Applied Sciences and Arts in Olten, Switzerland (SSW), and the Psychiatric Department at the Health Unit of Baixo Alentejo, Portugal (ULSBA), bring in their vast and extensive experience and knowledge of treating PG, and their skills in leading clinical evaluations. NOVA University of Lisbon (UNL) will bring in their expertise on testing and validating technological tools for health intervention, as well as economic evaluation and cost-effectiveness analysis. The Dutch insurer and funeral undertaker DELA Natura- en levensverzekeringen (DELA) will contribute with expertise from the innovation department, where they continuously seek new ways to support their clients around end of life, both online and offline. Finally, the Portuguese SME Sensing Future Technologies (SFT) will utilize their experience in marketing eHealth technologies for the Portuguese and international markets.

## Methods

### Core Technology Components

The core technology components in LEAVES are the mobile app Before You Leave (technology readiness level [TRL] 9, developed by DELA), the Livia Online Grief Program (TRL 7, developed by the UoB), and the natural language-based Virtual Agent Platform (TRL 7, developed by RRD). All modules will receive an accessible design and a clean user experience given the subject matter of coping with grief (furnished by NTH).

#### Before You Leave

After being notified about the LEAVES app by his caretaker, Frank downloads the LEAVES app on his tablet in order to leave Mary with memories. He opens the app and chooses a conversational agent to his liking: Peter. This agent questions Frank about defining moments in his life, his values, and wishes for after he passes away.

**Figure 2:**
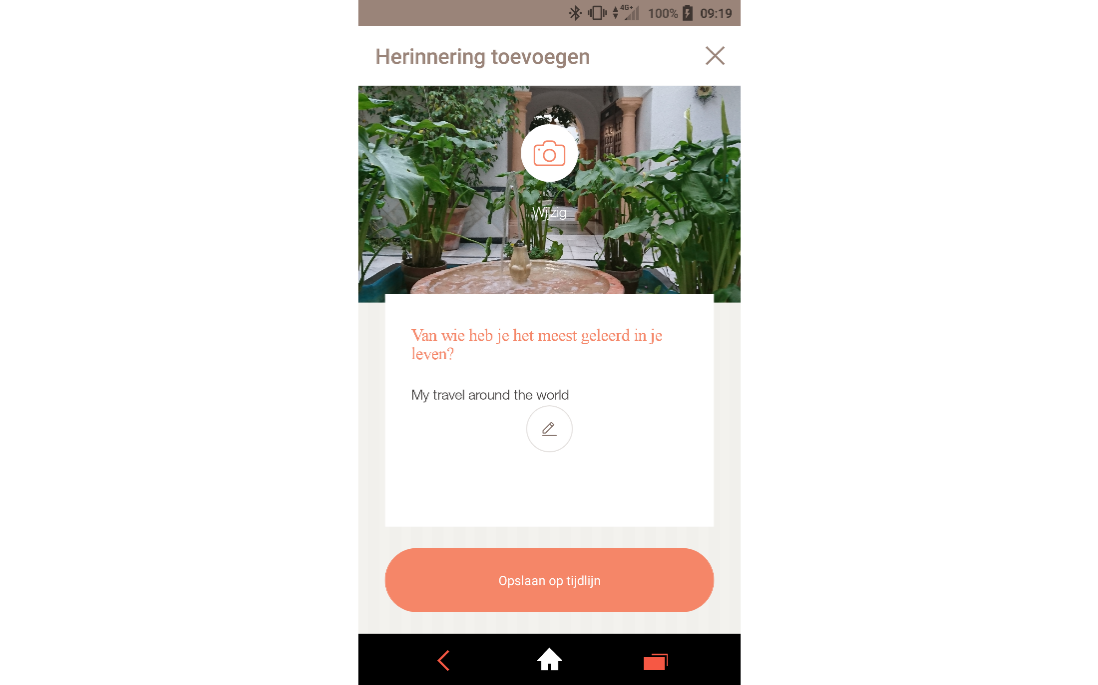
Before You Leave screen where a person is asked to reflect on whom they have learned most from in life.

The mobile app “Before You Leave” (Figure 2) offers those with a terminal condition the option to store memories, personal information, messages to relatives, and personal wishes about how to deal with personal affairs after passing away. The user is guided through a documentation process via an automatized chatbot and cue cards, each providing a question (out of a total of 500+ cues). The app allows users to store audio, pictures, and video for each question. After the event of death, relatives of the deceased can access this information in order to have a repository of personal information about the deceased and to be able to refer to the deceased’s wishes when faced with decisions about handling the deceased’s affairs. At the moment of writing, the Before You Leave service is not available anymore, as the value proposition turned out not to correspond with the envisioned end-users’ needs. The content and functionality of the service can be valuable for enriching the LEAVES service by allowing mourners to reflect on their life with their partner through the cues and other functionalities. This will be incorporated into the LEAVES service model and technology.

#### The LIVIA Online Grief Self-Help Program

Interested, Mary signs up [in LEAVES] using her tablet and is first asked a set of questions. By answering these questions, the LEAVES service learns what type of mourner Mary is, so that it can adapt its content to best connect with her.

**Figure 3:**
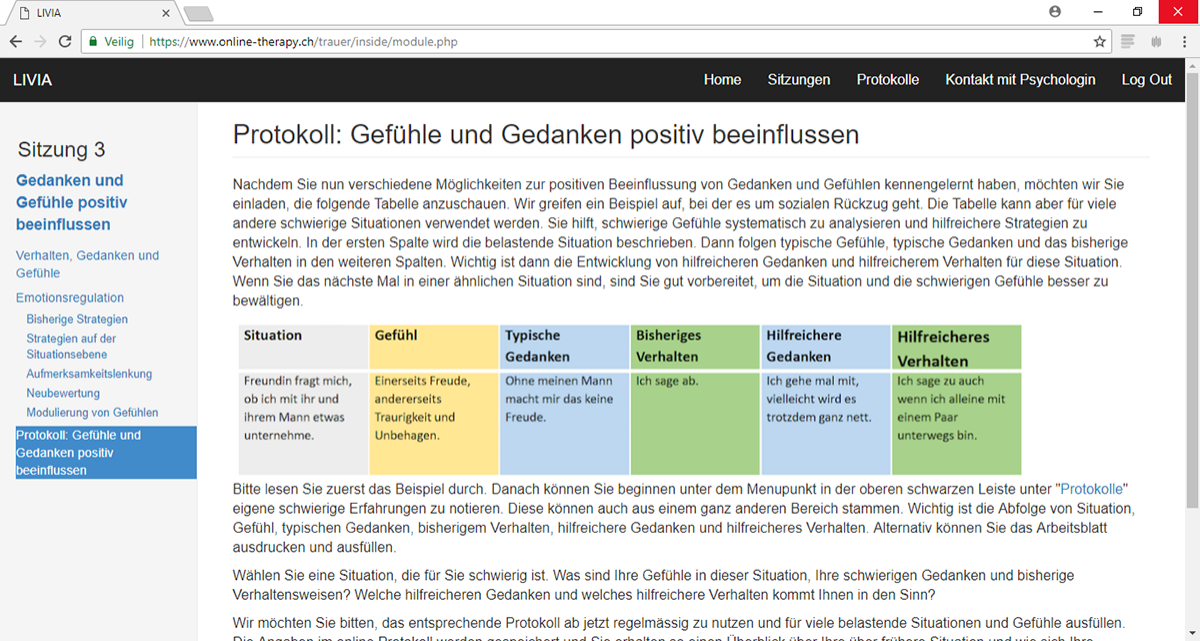
Online grief self-help program LIVIA.

The online self-help program LIVIA (Figure 3) addresses older adults who experienced divorce or marital bereavement greater than 6 months prior and who seek help for coping with PG symptoms, psychological distress, or adaptation problems in daily life. LIVIA has been developed and evaluated as a stand-alone guided self-help program [17,18]. The intervention is based on the task model of mourning [22] and the dual-process model of coping with bereavement [23]. LIVIA employs standard cognitive behavioral techniques for behavioral activation and modifying dysfunctional thoughts. In addition, writing tasks are used to clarify the meaning of the loss. Cognitive behavioral techniques are also used for identifying and activating intrapersonal and social resources, improve emotion regulation skills, and increase loss-related coping self-efficacy. This intervention aims to foster resilience, defined as the capacity to cope with the stressors related to the loss and adapt to a life without the spouse. A detailed description of the intervention can be found elsewhere [17,18].

LIVIA consists of 10 text-based sessions, and includes (1) information about interpersonal loss and an assessment of the current personal situation; (2) exposure and loss-oriented interventions, such as writing tasks for accepting memories and pain, and addressing unfinished business; and (3) resources and restoration-oriented interventions for fostering positive emotions and thoughts, self-care, creating a new life without the partner, and promoting positive social relationships. In its current form, guidance includes 1 short, weekly email. In addition, participants can contact their coaches via a contact button for further support. LIVIA has demonstrated significant improvements in reducing grief, depression, psychopathology, loneliness, and embitterment [17].

#### The RRD Virtual Agent Platform

Mary also interacts with the LEAVES service via a conversational agent. She chooses a virtual agent named Wendy from a set of options. Wendy informs Mary about the phases that she is going through, answers her questions, and asks her to do exercises (eg, reflecting on a difficult period). Wendy regularly presents Mary with memories or stories that Frank provided to his conversational agent, Peter. Every 2 weeks, Wendy also asks Mary how she is doing via a few short questions on the LEAVES smartphone app. From the conversations with Mary, Wendy infers Mary’s emotional state and adapts its content.

The RRD Virtual Agent platform is part of the back end of the LEAVES platform and consists of a set of tools and services that enables the creation and execution of dialogue-based natural interaction interfaces with end users. The platform is used to develop personal virtual agent companions in the context of eHealth apps that allow users to have tailored, dynamic conversations with a virtual agent. A core component of the platform is the WOOL Dialogue Framework, a simple, powerful dialogue framework for creating virtual agent conversations and completely open source licensed under an MIT License. More information and the tool itself can be found on the WOOL website [24]. The WOOL framework consists of 3 main components: (1) the dialogue language definition, (2) an easy-to-use editor for creating dialogue scripts, and (3) a set of tools to execute these dialogues. The WOOL Dialogue Framework was created in the context of the Council of Coaches project, a research and innovation project funded by the European Commission’s Horizon 2020 Research Programme.

**Figure 4:**
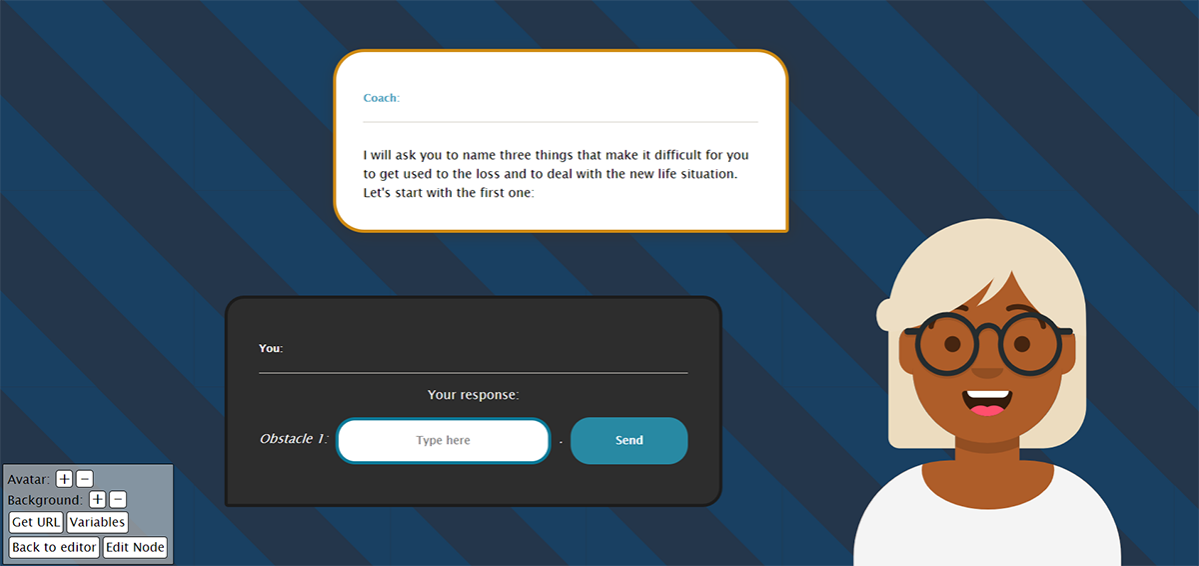
Example virtual agent "Anja" asking for input from the user on obstacles faced to deal with the loss of a partner.

The RRD Virtual Agent platform was designed to provide tailored motivational advice on various health domains and is grounded in theory on tailoring health communication [25] and providing motivational support in the physical activity promotion domain [26]. The screenshot in Figure 4 shows an example virtual agent, demonstrating how a simple natural language interaction is achieved using a text-based user interface and allowing the user to choose his/her replies from a pregenerated list. The core technology platform consists of dialogue authoring tools and a client–server architecture for executing the tailored dialogues.

The content in LEAVES will be offered by the virtual agent in “bite-sized” chunks and expressed in natural “conversational” style, making the contents of the service more accessible.

#### The LEAVES Platform Architecture

**Figure 5:**
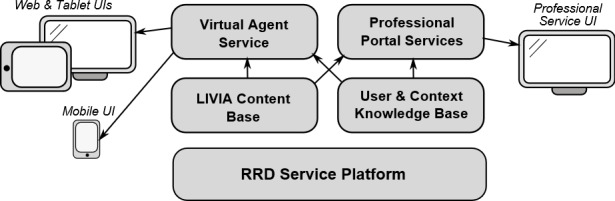
LEAVES (optimizing the mentaL health and resiliencE of older Adults that haVe lost thEir spouSe via blended, online therapy) service component overview. UI: user interface.

Figure 5 outlines the component overview for the LEAVES platform. The RRD Service Platform forms the basis for user management and secure data storage and access, and also provides APIs to the virtual agent service and professional portal services that serve the various core LEAVES user interfaces. Input to the platform is provided through the Before You Leave Service and LIVIA Content Base.

### Workplan

**Figure 6:**
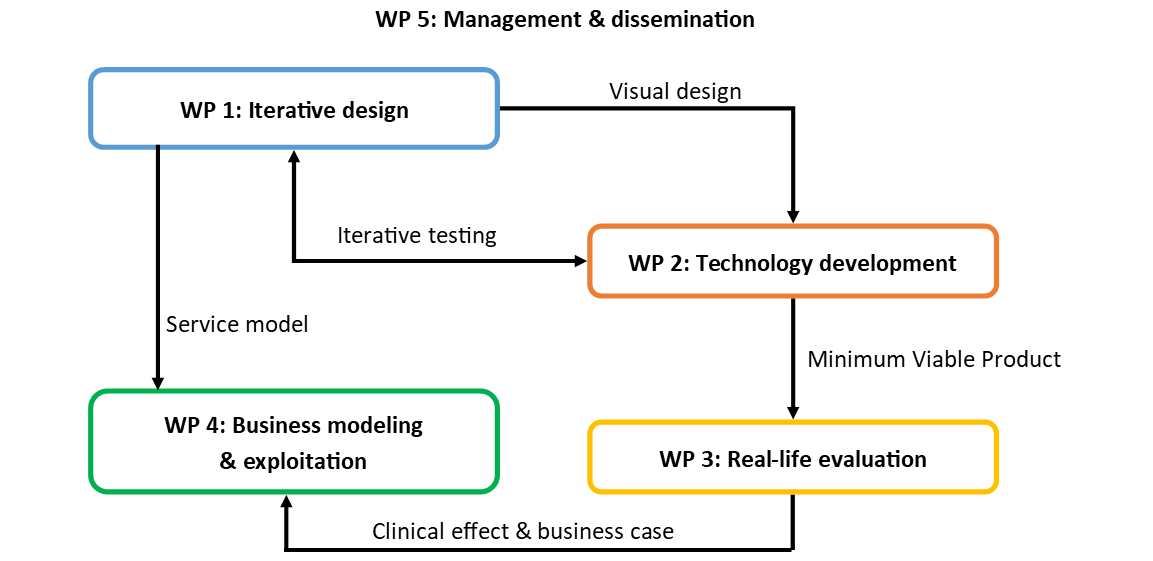
LEAVES (optimizing the mentaL health and resiliencE of older Adults that haVe lost thEir spouSe via blended, online therapy) work package (WP) overview.

The workplan of the LEAVES project is divided into the 5 work packages illustrated in Figure 6: (1) iterative design, (2) technology development, (3) real-life evaluation, (4) business modeling and exploitation, and (5) management and dissemination.

The first 2 years of the project will focus on the design and development of the LEAVES platform and its service model. These activities are mostly handled in work packages 1 and 2.

The objective of the first work package (ie, iterative design) is to make sure that the service, tools, and user interface design are in line with the expectations of all end-user groups of the LEAVES service. Several multidisciplinary design workshops will take place in which a wide array of techniques from user-centered design will be employed, including user story mapping, interviews, definition of “jobs to be done,” focus group discussions, information architecting, persona definitions, and card sorting for content aggregation. The results of these procedures will lead to first sketches and wireframes of the interface and its interactive experience. The user interface will be crafted with careful attention to accessibility standards, considering the specific needs of the elderly in user experience and user interaction. Iterative evaluations with different prototypes (with low, medium, and high fidelity) will be performed with example members of the target group. For the low- and mid-fidelity prototype evaluation, a series of fairly detailed mock-ups will be available, providing simulated interactive functionality and navigation. The prototypes will be presented to the users in a controlled laboratory environment, and the evaluations will be conducted in different iterative cycles with usability experts. Before the final pilot, an extensive usability study will be performed to make sure that all aspects are usable within the evaluation.

The second work package (ie, technology development) deals with all technology developments needed to realize a stable platform to be used in the real-life evaluation. To do so, the technology partners will work together setting working prototypes to maximize the impact of the iterative design work in the first work package. Technical and clinical partners will work together to translate the plain text content of LIVIA and Before You Leave into dialogue-based text. This work package will also deal with the personalization of the content to specific end users based on user profiles. A continuous personalization of the service will be performed as a response to the emotional assessment performed in the platform. Last but not least, this work package will ensure that the content system will be designed with a “privacy by default” approach. In the LEAVES project, we will go beyond the state of the art in privacy design and will include a personal data control interface that allows older adults (with relatively undeveloped technological skills and low awareness of privacy control) to control the access and storage of their personal data. Given the sensitive nature of the treatment and the data that are collected about the individual who uses the LEAVES service, ensuring privacy and control over personal data has top priority.

The third and last year of the project will be mostly dedicated to real-life evaluation, refinement of the business model, and exploitation. The third work package (ie, real-life evaluation) focuses on the evaluation of the clinical and economic potential of the LEAVES service by conducting a longitudinal real-life evaluation across 3 countries: Portugal, the Netherlands, and Switzerland. This work package concerns the development of the study protocol as well as the clinical evaluation (in Portugal and Switzerland) and acceptance of technology and service design (in the Netherlands). Finally, an economic evaluation will be performed by determining the differences in costs and the health benefits that result from the use of LEAVES.

The clinical evaluation will involve older adults (65+) who express the need for help in mourning their spouse and will focus on well-being (eg, a decrease in grief symptoms measured with the Texas Revised Grief Inventory [27]; loneliness, as assessed with the De Jong Gierveld Short Scale for Emotional and Social Loneliness [28]; and improved quality of life measured with the Satisfaction with Life Scale [29]) and end-user satisfaction. Results will be compared with those obtained in the same timeframe from people that are “regular mourners” (in The Netherlands), and in a waiting control group (in Switzerland and Portugal). Next, close ones to older adults and care professionals will be consulted on potential effects of the service, to gather a comprehensive overview. Exit strategies will be put in place for participants who need additional care or want to stop using the service. The objective of the economic evaluation is fourfold and will aim to assess the financial consequences from implementing the LEAVES service. It will (1) estimate the change in symptoms averted with the implementation of LEAVES; (2) evaluate the costs associated with the implementation of LEAVES; (3) evaluate the avoided costs related to minimization of the grief symptoms; and (4) compare the costs of LEAVES with the benefits of implementing it, in terms of improved health.

The fourth work package (ie, business modeling and exploitation) concerns the development of a business model, integrated and supported on the evidence from the field, as well as a go-to-market strategy and commercial exploitation of the project output.

The fifth and final work package (ie, management and dissemination) concerns project management and ensures that the project will be executed on time, within budget, and with outstanding quality. Within this work package a dissemination and communication strategy is also defined and executed. Finally, the ethical guidelines for the project will be established, including procedures for medical ethical permission and informed consent, as well as project-wide methods for data handling (in a General Data Protection Regulation [GDPR]-compliant manner).

## Results

The project runs from February 1, 2020, to January 31, 2023. Results of the project will be reported continuously following a dissemination and communication plan developed in the first year of the project. The dissemination plan follows annual goals: (1) create awareness about the concept, (2) stimulate involvement, and (3) facilitate exploitation. End users, potential lead users, the business community, researchers, and the general public are foreseen as prospective target groups.

## Discussion

After 3 months, Mary feels ready to move on. This is confirmed by an online screening administered by Wendy that assesses Mary’s resilience, self-sufficiency, loneliness, and happiness. Mary unsubscribes from the LEAVES service and deletes the app from her phone.

In this proposal, we have described the development, evaluation, and exploitation of an online service that has the primary aim to prevent or treat PG among older adults who have lost their spouse. The project will innovate by developing a blended, virtual agent–based bereavement support program, in contrast to existing grief programs that rely mostly on text-based interventions. Robust monitoring mechanisms for detecting the onset and escalation of PG will be developed. Finally, the technology will be implemented and tested in 3 countries to ensure a pleasant user experience, gauge its effect, and determine its cost-effectiveness. Guaranteeing follow-through, the LEAVES consortium will work toward successful exploitation of the service.

## Appendix

Multimedia Appendix 1 Review of the LEAVES proposal.

## Abbreviations

DSM: Diagnostic and Statistical Manual of Mental Disorders
ICD: International Classification of Diseases
LEAVES: optimizing the mentaL health and resiliencE of older Adults that haVe lost thEir spouSe via blended, online therapy
NFE: Dutch National Foundation for the Elderly
NTH: Swiss SME Nothing AG
PG: prolonged grief
RRD: Roessingh Research and Development
SFT: the Portuguese SME Sensing Future Technologies
SSW: School of Social Work of the University of Applied Sciences and Arts in Olten, Switzerland
TRL: technology readiness level
ULSBA: Psychiatric Department at the Health Unit of Baixo Alentejo, Portugal
UNL: University of Lisbon

## References

[1] Prigerson HG, Frank E, Kasl SV, Reynolds CF, Anderson B, Zubenko GS, Houck PR, George CJ, Kupfer DJ (1995). Complicated grief and bereavement-related depression as distinct disorders: preliminary empirical validation in elderly bereaved spouses. Am J Psychiatry.

[2] Molina N, Viola M, Rogers M, Ouyang D, Gang J, Derry H, Prigerson HG (2019). Suicidal Ideation in Bereavement: A Systematic Review. Behav Sci.

[3] Stahl ST, Schulz R (2014). Changes in routine health behaviors following late-life bereavement: a systematic review. J Behav Med.

[4] Stroebe M, Schut H, Stroebe W (2007). Health outcomes of bereavement. Lancet.

[5] Parisi A, Sharma A, Howard MO, Blank Wilson A (2019). The relationship between substance misuse and complicated grief: A systematic review. J Subst Abuse Treat.

[6] Ennis J, Majid U (2019). Death from a broken heart?: A systematic review of the relationship between spousal bereavement and physical and physiological health outcomes. Death Stud.

[7] Lundorff M, Holmgren H, Zachariae R, Farver-Vestergaard I, O'Connor M (2017). Prevalence of prolonged grief disorder in adult bereavement: A systematic review and meta-analysis. J Affect Disord.

[8] Kersting A, Brähler E, Glaesmer H, Wagner B (2011). Prevalence of complicated grief in a representative population-based sample. J Affect Disord.

[9] Newson RS, Boelen PA, Hek K, Hofman A, Tiemeier H (2011). The prevalence and characteristics of complicated grief in older adults. J Affect Disord.

[10] Bonanno G, Wortman CB, Lehman DR, Tweed RG, Haring M, Sonnega J, Carr D, Nesse R (2002). Resilience to loss and chronic grief: a prospective study from preloss to 18-months postloss. J Pers Soc Psychol.

[11] Aoun SM, Breen LJ, Howting DA, Rumbold B, McNamara B, Hegney D (2015). Who needs bereavement support? A population based survey of bereavement risk and support need. PLoS One.

[12] Ott CH, Lueger RJ, Kelber ST, Prigerson HG (2007). Spousal bereavement in older adults: common, resilient, and chronic grief with defining characteristics. J Nerv Ment Dis.

[13] Eisma MC, Boelen PA, van DBJ, Stroebe W, Schut HAW, Lancee J, Stroebe MS (2015). Internet-Based Exposure and Behavioral Activation for Complicated Grief and Rumination: A Randomized Controlled Trial. Behav Ther.

[14] Litz BT, Schorr Y, Delaney E, Au T, Papa A, Fox AB, Morris S, Nickerson A, Block S, Prigerson HG (2014). A randomized controlled trial of an internet-based therapist-assisted indicated preventive intervention for prolonged grief disorder. Behav Res Ther.

[15] Van der Houwen K, Schut H, Van den Bout J, Stroebe M, Stroebe W (2010). The efficacy of a brief internet-based self-help intervention for the bereaved. Behav Res Ther.

[16] Wagner B, Knaevelsrud C, Maercker A (2006). Internet-based cognitive-behavioral therapy for complicated grief: a randomized controlled trial. Death Stud.

[17] Brodbeck J, Berger T, Biesold N, Rockstroh F, Znoj HJ (2019). Evaluation of a guided internet-based self-help intervention for older adults after spousal bereavement or separation/divorce: A randomised controlled trial. J Affect Disord.

[18] Brodbeck J, Berger T, Znoj HJ (2017). An internet-based self-help intervention for older adults after marital bereavement, separation or divorce: study protocol for a randomized controlled trial. Trials.

[19] DELA Innovation Lab (2018). Before You Leave App [Internet].

[20] Bickmore TW, Utami D, Matsuyama R, Paasche-Orlow MK (2016). Improving Access to Online Health Information With Conversational Agents: A Randomized Controlled Experiment. J Med Internet Res.

[21] Op den Akker H, Op den Akker R, Beinema T, Banos O, Heylen D, Bedsted B, Pease A, Pelachaud C, Salcedo V, Kyriazakos S, Hermens H (2018). Council of Coaches - A Novel Holistic Behavior Change Coaching Approach. http://www.scitepress.org/DigitalLibrary/Link.aspx?doi=10.5220/0006787702190226.

[22] Worden J (2018). Grief Counseling and Grief Therapy: A Handbook for the Mental Health Practitioner (5th ed.).

[23] Stroebe M, Schut H (1999). The dual process model of coping with bereavement: rationale and description. Death Stud.

[24] Roessingh Research and Development (2020). The WOOL Platform.

[25] op den Akker H, Jones VM, Hermens HJ (2014). Tailoring real-time physical activity coaching systems: a literature survey and model. User Model User-Adap Inter.

[26] Op den Akker H, Cabrita M, Op den Akker R, Jones VM, Hermens HJ (2015). Tailored motivational message generation: A model and practical framework for real-time physical activity coaching. J Biomed Inform.

[27] Faschingbauer T (1981). The Texas Inventory of Grief--Revised.

[28] Gierveld JDJ, Tilburg TV (2016). A 6-Item Scale for Overall, Emotional, and Social Loneliness. Res Aging.

[29] Diener E, Emmons RA, Larsen RJ, Griffin S (1985). The Satisfaction With Life Scale. J Pers Assess.

