# Supplementary material for: LEAVES (optimizing the mentaL health and resiliencE of older Adults that haVe lost thEir spouSe via blended, online therapy): Proposal for an Online Service Development and Evaluation
Source: JMIR Res Protoc. 2020 Sep 8;9(9):e19344. doi: 10.2196/19344 (PMC7509633; doi:10.2196/19344)
Supplement: Multimedia Appendix 1 [file resprot_v9i9e19344_app1.pdf]

**The Active Assisted Living Programme**

**Evaluation Summary Report (ESR) -**

**Evaluation-call-2019**

# PROPOSAL: optimizing the mentaL health and resiliencE of older Adults that haVe lost thEir spouSe via blended, online therapy

**aal-2019-6-168-CP**

## 1. RELEVANCE AND SCOPE — 4 / PASSED

The proposal clearly addresses the Call challenge, as it intends to provide an ICT-based solution to support active, healthy and independent living for older adults. It is aligned with the AAL application areas Vitality & Abilities and Health & Care. LEAVES intends to develop an online grief platform aiming at preventing Prolonged Grief (PG). Users can either start the service to store memories online, a process that is guided by an assistant or, in case the partner has passed, interact with the platform and be supported in the process of mourning. Additionally, the system detects symptoms of depression and provides several options for help.

The proposal defines clearly the markets it is targeting, focusing on both markets, private consumer market as well as regulated markets, although in the market section it is clearly pointing to regulated actors (e.g. psychological counsellors, psychiatric hospital departments, mental health organizations, medical insurers, municipalities, elderly organizations).

The sample scenarios explain how the system works in an understandable way. The implementation scenario is convincing and realistic, the solution is desirable for the targeted end-users, as mourners often develop Complicated Grief (CG) or Prolonged Grief disorders. Furthermore, the proposal highlights that the development of PG has been found to trigger many mental and physical problems, including poor sleep, change in smoking and eating habits, cardiovascular problems, functional disability, depression, loneliness, and suicidal tendencies. The issue of loneliness is extremely relevant. Also, the challenges faced by end-users in the scenario are common to a group of older people across Europe and beyond. The proposal also depicts a good match between end user requirements and the proposed ICT solution, as the anticipated applications demonstrate potential to effectively address the challenges that are illustrated in the example scenario. There is a clear indication of health and social needs exemplified in the proposal, envisioning a realistic set of prerequisites for achieving the described benefits for senior mourners. Regarding the size of the chosen segment, the number is consistent (case of deaths) – loss of a spouse is a frequent occurrence in later life -, with a tendency to increase because of the aging society.

The rationale for this project is set out clearly in terms of aims and activities planned. Also, the proposal demonstrates that it has a very good understanding of the market audience that is being targeted, and it provides convincing statements that it will be able to identify and to reach the targeted groups. Its Unique Selling Point (USP) is that it provides “a low-threshold, engaging, self-service mourning program” (p. 26). The service will soften the mourning process, prevent depression and/or social isolation, strengthens widower/widow’s resilience, and quickens a return to societal participation. These effects tackle the challenges identified in the example scenario with a clear focus on the stated rationale of the project.

The identified novel elements are well-targeted to address the stated objectives of the project, offering an original personalized solution by dynamically adapting the contents, based on regular emotional monitoring. The anticipated system is well aligned with the state-of-the-art and available services (including Before You Leave, the Livia Online Grief Program and the RRD Virtual Agent Platform), presenting a clear added value if compared to the currently used solutions from an individual and societal point of view, integrating and improving available features (offering a low entry-barrier program and a natural and easy interaction guided by a friendly conversational agent, well grounded in scientific theory and clinical expertise).

LEAVES intends to make use of Artificial Intelligence (AI), and to introduce a conversational agent. It also implements a range of existing solutions: Livia, a program that focuses on guiding mourners during marital bereavement via a self-service online program, and the “Before You Leave service”, a program that enables users to collect memories. The proposal is therefore in line with the state-of-the-art technology.

The proposed ICT solution has overall sound potential to be commercialised, considering the anticipated market relevance of the envisaged systems, the appropriate business nature of the involved commercial partners and the clear indication of a commercial approach, although evidence of the willingness to pay is somewhat limited and leaves some risks in what regards the commercial success. In terms of freedom to operate, IPRs are not fully clearly allocated, in particular in connection to foreground development (representing a potential barrier for an effective commercial deployment). The envisioned business models have reasonable chances to be well received by target end-users, with a sufficiently demonstrated and anticipated cost-effectiveness of the proposed system.

The proposal presents a comprehensive market analysis showing a holistic view on the market and its participants. The provided reports support the assumption that the proposed ICT solution is likely to be commercially successful. Even if the proposed

solution does not have an effective ground-breaking character, there is sufficient novelty to provide a fair unique selling point to the target audience.
Key Performance Indicators (KPI) are presented in a table (p. 8) and take into account multiple dimensions (impact on users, impact on market, scientific impact, etc.); they are organised according to categories and most of them are provided with measurable goals. The chosen KPIs are relevant to the project rationale and activities, and they will enable to identify clearly if the project is performing well. Furthermore, they do match the proposed benefits from the system, as they mirror all relevant aspects of the project. The commercial potential for the envisaged ICT solution is identified through the proposed KPIs and the ways in which performance indicators will be measured are appropriate. The trial period is likely to enable the project to measure the KPIs (although missing a direct correspondence between KPIs and respective Tasks and Deliverables).

## 2. IMPLEMENTATION - QUALITY OF PROPOSED SOLUTION AND WORKPLAN — 4 / PASSED

The proposal provides a table that shows at a glance that all kinds of end-users, primary (older adults), secondary (family, relatives) and tertiary end-users (potential lead users, care professionals) are involved throughout the project, including the evaluation of process and results (table 2, End-user involvement in the LEAVES project, p. 9/10). The end-users involvement approach, project objectives and example scenario are consistent, although lacking a fully structured path and methodology to ensure the active participation of secondary end-users.
The anticipated approach to end-users' involvement follows established methods, namely human-centred design and collaborative design (co-design). The latter focuses on endpoints that are service model design, usability and user experience (UX) design, business model design, clinical and business evaluation. These methods support the fact that perspectives of end-users are considered in detail. Table 2 shows that the proposal is seriously considering end-users involvement, as end-users are involved in a wide range of project tasks, including evaluation of process and results.
This attitude is mirrored in the work packages, where especially work package 3 is dedicated to Real-Life Evaluation. WP3 is, with 79.5 person months, the work package with most of the efforts. A clinical evaluation, demonstrating the impact on older adults, is planned to take place within clinical settings. The focus is sufficient also on personal aspirations, satisfaction and self-esteem and not only limitations, building effectively on specific hypotheses to be tested and variables and outcomes to be assessed, primarily focused on quantitative parameters, relevant to the recognition of a well -substantiated, structured and detailed presentation of end-users' needs and expectations.
There is an overall sound consistency between the end-users' involvement approach and project objectives and the example scenario.
However, a recruitment strategy is not clearly outlined in the proposal, although under T3.2 Clinical evaluation and technology acceptance it is mentioned that recruitment of participants will be the responsibility of the partners NFE, DELA and ULSB – these are the user organisations in the consortium - in each of the respective countries. The proposal defines well which type and number of end-users will be included. Size indications for the technical pilot and deployment tests are provided, yielding achievable and accordingly ambitious targets (315 end users). The testing activity will be implemented in the involved end-users' living environments in three test countries. Moreover, the ethical approach supporting the involvement of end-users is appropriately defined.
The proposal sets out a convincing case that it will create a realistic environment to test the prototype, in particular for the pilot tests, and on a sufficiently identified path to ensure testing users' involvement, matching closely the example scenario.
Key stakeholders in the three participating countries are intended to be included in all phases of the project and in the market introduction (planned within one year after the end of the project). Stakeholders that will be responsible for deployment, implementation and commercialisation are going to be involved in all work packages with an amount of person months that is adequate to have a substantial influence over the relevant work plan activities. However, the proposal lacks a fully effective path to ensure an effective involvement from the so called "lead users" (psychological counsellors, psychiatric hospital departments, mental health organizations, medical insurers, municipalities).
Overall, the proposal is supported by an overall sufficient engagement and influence of critical stakeholders in all project phases. Industrial and implementation partners are engaged in a substantial level of work leading up to market launch. The effort of industrial and business partners is more than 60% of the person months total. The proposal intends to reach a significant engagement and influence also from critical and "lead" stakeholders outside the consortium.
The proposal describes in detail the existing components/modules that will be connected to a novel solution. The core technology development focuses on the integration of mobile applications like "Before you leave", or the "Livia Online Grief Program" into the so-called Virtual Agent Platform. This platform is described as a set of tools and services, "that enables the creation and execution of dialogue-based natural interaction interfaces with end-users" (p. 12). It handles user management, secure data storage and access. The service components of the solution are graphically presented (fig. 7, LEAVES Service Component Overview, p. 13). From the reports on how the technology is built and which technical models can be used, it is evident that the planned technology development and deployment is tangible and convincing. Also, the proposal includes a clear description of the technical work to be undertaken, the challenges that will be addressed by the development work, and the steps to be undertaken throughout the technical work plan.
The proposal sets out appropriately within task 1.2 how it intends to design simple and intuitive interfaces, with a balanced effort devoted to such tasks and a sufficient description of the technical architecture, supported by a clear graphical description of the solution, key processes functions and relationships, indicating a sound technical competence of the team, their appropriate development approach and their understanding of the challenges to be addressed. Functionalities of the final system are clearly presented as well, referencing also the relevant standards, communication and dialogue structure protocols (e.g. REST APIs and documented JSON formats).

The proposal sets out with specific information the steps by which end-user feedback will actively have an influence on the shape of the technology development, through iterative incremental development steps designed to influence and fine-tune the system and its functionalities.
The proposal demonstrates a clear understanding of the Technology Readiness Level (TRL) of the ICT solution at the start, throughout the project and at the end of project development. All modules that will be combined are provided with their TRL (see page 3, Unique selling proposition), with the "Before You Leave" program with the highest level 9 (the others 7).
Privacy is an issue that is handled in a novel way, going beyond the state of the art in privacy design. The proposal gives top priority to ensuring privacy and control over personal data, applying the General Data Protection Regulation (GDPR), although limited details are provided on how security issues will be technically addressed. In order to make the privacy-related features of the solution a selling point, a personal data control interface will be developed. This application will allow older adults to control the access and storage of their personal data, even if they have relatively undeveloped technological skills (p. 13). Privacy is part of work package 2, Technology Development, as T2.4, Privacy and control, connected to D2.2 Designing for Privacy Control (M24).
The proposal indicates that the partnership has a good knowledge of standards, certification, interoperability and universal design.

## 3. IMPLEMENTATION - QUALITY OF CONSORTIUM AND PROJECT MANAGEMENT — 5 / PASSED

The partnership is very well composed, consisting of four business partners, Small Medium Enterprises as well as a Large Enterprise, three user organisations and two research institutes. Three partners are located in The Netherlands, three in Switzerland and three in Portugal. The expertise domains of the consortium members is visualised in a table (p. 14), and these domains not only comprise expertise like project management, end-user testing etc., as usual, but also the expertise "Grief and mourning", Clinical evaluation, Business case evaluation etc. This table shows that the partners have complementary skills and experience, the combination of all is likely to support a successful fulfilment of the project including commercialisation. The collaboration of European partners is well justified and needed, to accommodate the various skills, confront with national regulations and realities, deploy competences and compare different national scenarios for system testing, preparing for an international market approach.
The detailed review of the work plan indicates that each of the identified activities has an experienced and capable leading organisation available. Not only the work packages as a whole have an experienced leader, but also each task itself.
Work plan, distribution of effort and clear responsibilities are likely to support a good cooperation of the partners. The budget shows a good proportion of work and financial resources. Suitable rules for conflict resolution are elaborated beforehand. Together with the very well-designed management, there is evidence that problems will easily be identified and swiftly managed. In order to facilitate smooth collaboration, committees with clear responsibilities are established, like a Steering Committee, an End-user Committee, a Technical Committee and an Exploitation Committee as well as an Advisory Board, staffed with General Practitioner representatives, eMental health experts and business representatives (p. 16).
All resources requested are clearly set out and in line with expected results and impact, with the presence of a clear indication and allocation of resources and roles among the partners and of a sound level of general detail in the content of the various technical tasks.
Following the description of the consortium members, it is evident that access to the most required resources exist at the start of the project, including the right personnel, networks of stakeholders and end users.
Risk assessment is part of project management (WP5), Deliverable 5.1, Quality, Risk and IPR Management Procedures (M3), where a risk inventory and risk management strategy are to be developed. A first risk inventory is part of the proposal and is presented in a table, that shows risk description, likelihood (1-5), impact (1-5), action (avoid, mitigate, transfer, accept), mitigation plan and responsible partner. This table indicates a very high awareness of the partners on risks and is a very good foundation for risk management. The risks that it describes are consistent with the project objectives.
The proposals made for managing risks are proportionate and effective. They are clear indicators that risks will be reviewed actively and managed well. This assessment is underlined by the statement in the proposal that risk assessment will be part of consortium meetings that are scheduled to take place two times a year, where the risk assessment plan will be updated regularly. The risk register is project specific and precise; moreover, mitigation actions are concrete, actionable and provided with a responsible partner.
The proposed quality assurance and control procedures for deliverables are sufficiently adequate to ensure that eventual poor performance by any of the consortium members is picked up swiftly and managed actively, although a dedicated approach towards a review of project activities and deliverables is missing.
There is an initial assessment of risks and opportunities related to the market introduction of the solution, although with a quite simplified approach and not presenting fully effective contingency provisions in case of difficult market entrance and acceptance.

## 4. IMPACT - POTENTIAL IMPACT OF PROPOSED SOLUTION ON QUALITY OF LIFE — 4 / PASSED

Considering that the loss of a spouse can lead to prolonged grief, where grief symptoms occur longer and may result in mental and physical problems, like poor sleep, cardiovascular problems, depression and loneliness, the proposed solution, with its broad offer of personalisble support, is an opportunity to contribute to improve the quality of life of bereaved older adults, the primary end-users, by softening the mourning process, preventing depression or social isolation, strengthening resilience and wellbeing. The solution provides therapeutic strategies to reduce the distress and emotional suffering, thus increasing the psychological well-being. It assists the end-users to activate resources in daily life and promotes building positive relationships.
For the secondary end-users, family, neighbours, friends it is acknowledged that they are concerned about the mourner's situation but often do not know how to deal with the emotional status. They would like to give support, but often do not know how. The

proposed solution can help reducing their stress, worries and emotional care burden. They can rely on the service as the status of the mourner is monitored automatically, providing help when needed. Therefore, the project outcomes are likely to deliver also benefits to the quality of life for secondary end-users, reducing stress and emotional care burden for close ones, friends and neighbours, and the proposal is well designed to appreciate accordingly the extent of such benefits, with assessment plans focused also on secondary end-users.

Current service models are described in the proposal to be curative and utilise mainly face-to-face psychological counselling sessions. It is stated that preventive services are scarce. The proposal identifies the appropriate service models in the domain targeted and the limitations of the actually available solutions.

The service model of LEAVES will introduce a model to prevent PG, a self-service approach towards preventing or treating PG, and it will enable novel means of introducing care to mourning persons. The result could be that the existing care path will be altered; yet, the proposal provides a convincing argument that the "old" service models will be positively affected by the solution. Also, the solution opens new opportunities for services for general practitioners, psychological counsellors as well as organisations for the elderly, who could offer the services as part of their own. The proposal demonstrates that the consortium has a good understanding of the market landscape, and the service models are identified clearly and realistically. The proposal develops several ways to show how the solution can be integrated with service delivery, for example into existing medical workflows.

Further, the proposal sets out a clear case that the envisaged solution will be capable of integration with relevant operational environments. The solution is clearly expandable, reusable and therefore scalable, building on a good understanding of the market landscape. Service models, underpinning delivery of the proposed ICT solutions, are clearly and realistically identified.

It cannot be assessed if all relevant ethical issues are correctly addressed, as they are part of work packages, especially work package 5, Management and Dissemination, here T5.3 Ethics and Data Management, in connection with D5.4 Ethical and data management guidelines (M6), but with a limited level of detail provided in the proposal.

The proposal clearly describes the expected social impact of the solution, mentioning that it promotes social inclusion and active engagement, and that it will lead to reduced burden of care for informal caregivers. A wider benefit is lower costs for mental care which means savings for the care system.

The proposal has developed a sound strategy to manage the departure or loss of end-user participants during the project, as it mitigates the risk of under-inclusion by recruiting more end-users and stakeholders than necessary. A drop-out percentage of 15 % is anticipated. However, no other steps are planned in case the drop-out rate is higher than anticipated.

The proposal provides substantial evidence that it has identified strategies to minimise blockages to end-user participation, and a good strategy for ending the participation of involved seniors.

## 5. IMPACT- POTENTIAL IMPACT OF PROPOSED SOLUTION ON MARKET DEVELOPMENT — 4 / PASSED

The proposal plans the market entry within one year after the project has ended. Work package 4 deals with Business Modelling and Explanation. Here and in other parts of the proposal the activities necessary to reach the market within this time are clearly described.

The proposal gives a very good insight into the product and how it will look like at the end, supported by graphics and figures, for example with a figure illustrating the LEAVES Value Chain (p. 6).

The analysis of competing offers and existing service models is complete. Relevant existing programs (e.g. Before you leave) are duly referenced, although not making fully clear the effective level of usage of such applications. The proposed ICT solution has the potential to fit the gaps identified within the market landscape it is targeting.

The proposal illustrates that the solution could easily implement similar mental health interventions, for example for dealing with divorce. The system could therefore be useful for differing target groups and so increase its chances of successful exploitation. The system can be extended to younger target groups as losing their partner will result in grief regardless of age. Also, the proposal identifies appropriately how the project outcomes could be deployed at scale within other market environments than those targeted, in particular involving not only the primary end-users (seniors) but also secondary and tertiary targets.

The proposal presents a convincing number of people that will constitute the potential market. However, the proposal does not discuss appropriately the fact that – and this is predictable – a loop of constant new customers must be found, as mourners will probably leave the program as soon as they feel fit for life again. There is a sufficiently convincing description of why the targeted users or customers will want to use or buy the solution, including explicit cost-benefits assumptions, also for care organizations.

The proposal has a clear view of the current and anticipated market and it sees that its "highly innovative approach in terms of technology and service model design" (p. 26) will ensure that this competitive edge will "retain for the coming five years" (p. 26). There is a realistic understanding of the market landscape, including stakeholders in the value chain. The estimation of the market size is convincing. Willingness to pay from the regulated market actors is also sufficiently well motivated.

A tentative business model is described in the proposal that forms the starting point of the development of the final business model that will be developed within work package 4 and already moves beyond a generic model, as it includes project-specific features. The relevant elements of the business strategy are present, including clear business and commercial roles of the partners.

Business Modelling and Exploitation is the name of work package 4, all of its tasks deal with business model development and exploitation and will lead to related deliverables. With 56 person months (20 % from total) the resources allocated to these tasks are appropriate; in addition, WP1 and WP3 also contribute to exploitation as they deliver a service model and a business case.

The proposal sets out a strategy for developing the business model during the project period, for example organising three partner workshops and focus groups to define the value proposition, with the aim to secure the commitment of three customers that will buy the solution when ready. The time to market and the proposed timeline are overall credible, with a sound focus on business modelling and pricing policy, other than clients' quantification.

The costs are set out well and realistically.

Within the partnership, roles are clearly defined from the beginning and with relation to the partners’ expertise. It is clear which partner is responsible for commercialisation and market entry. The initial value-for-money proposition is credible, as are the financial projections for the project in the future (revenue estimations). External stakeholders required for market success are well identified, although missing a fully structured plan to ensure their engagement.
The proposal includes a dissemination plan, detailed in a table (Communication plan of LEAVES, p. 29), where target groups, key messages, channels and also the frequency are clearly set out. With its three distinct yearly goals (create awareness, focus the awareness and maintain awareness) shows a good initial understanding of the differing audience groups for dissemination and marketing, also for the relevant messages and channels to be used to target the audience groups. Dissemination and communication are part of T5.2 in work package 5, Management and Dissemination, with the related Deliverable D5.2 Dissemination & communication plan, which is planned as a living document and will be updated yearly (M6, M12, M24, M36). The initial dissemination plan presented in the proposal includes appropriate quantitative targets (e.g. number of events, of articles to be published, etc.), facilitating monitoring its progress and appreciating its effective potential impact.
Intellectual Property Rights (IPR) are addressed sufficiently, as the consortium will apply rules for participation in European research projects, and IPR will be part of the Consortium Agreement (CA), where IPR management for the duration of the project is appropriately specified. Background knowledge is well identified. The approach is able to ensure a fair protection to consortium members and supports the chances of successful exploitation of the project outcomes. IPRs are also part of work package 5, Management, D5.1. However, the need for 'freedom to operate' is not fully apparent as IPR management does not make fully clear how the shared ownership of some key foreground results will be effectively managed for an effective commercial deployment.

## 6. REMARKS — NOT RATED

Reflecting what was already commented under the various assessment criteria, the following remarks and recommendations are given:
• a more structure path and methodology should be included, to ensure the active participation of secondary end-users and of the so called “lead users” (psychological counsellors, psychiatric hospital departments, mental health organizations, medical insurers, municipalities);
• the recruitment strategy for end-users’ participation to the piloting should be further outlined;
• further details should be provided on how security issues will be technically addressed;
• more details should be provided on ethical issues’ management and provisions;
• effective steps should be included, to be taken in case the drop-out rate from piloting results is higher than anticipated;
• the proposal should make clearer how the shared ownership of some key foreground results will be effectively managed for an effective commercial deployment and effective IPR management.

**TOTAL SCORE: 21**
